# Developing and Applying a Formative Evaluation Framework for Health Information Technology Implementations: Qualitative Investigation

**DOI:** 10.2196/15068

**Published:** 2020-06-10

**Authors:** Kathrin Cresswell, Robin Williams, Aziz Sheikh

**Affiliations:** 1 Usher Institute The University of Edinburgh Edinburgh United Kingdom; 2 Institute for the Study of Science, Technology and Innovation The University of Edinburgh Edinburgh United Kingdom

**Keywords:** health information technology, evaluation, sociotechnical

## Abstract

**Background:**

There is currently a lack of comprehensive, intuitive, and usable formative evaluation frameworks for health information technology (HIT) implementations. We therefore sought to develop and apply such a framework. This study describes the Technology, People, Organizations, and Macroenvironmental factors (TPOM) framework we developed.

**Objective:**

The aim was to develop and apply a formative evaluation framework for HIT implementations, highlighting interrelationships between identified dimensions and offering guidance for implementers.

**Methods:**

We drew on an initial prototype framework developed as part of a literature review exploring factors for the effective implementation of HIT. In addition, we used qualitative data from three national formative evaluations of different HIT interventions (electronic health record, electronic prescribing, and clinical decision support functionality). The combined data set comprised 19 case studies of primarily hospital settings, and included 703 semistructured interviews, 663 hours of observations, and 864 documents gathered from a range of care settings across National Health Service (NHS) England and NHS Scotland. Data analysis took place over a period of 10 years and was guided by a framework informed by the existing evidence base.

**Results:**

TPOM dimensions are intimately related and each include a number of subthemes that evaluators need to consider. Although technological functionalities are crucial in getting an initiative off the ground, system design needs to be cognizant of the accompanying social and organizational transformations required to ensure that technologies deliver the desired value for a variety of stakeholders. Wider structural changes, characterized by shifting policy landscapes and markets, influence technologies and the ways they are used by organizations and staff.

**Conclusions:**

The TPOM framework supports formative evaluations of HIT implementation and digitally enabled transformation efforts. There is now a need for prospective application of the TPOM framework to determine its value.

## Introduction

Health systems worldwide are prioritizing the implementation of health information technology (HIT) in the quest to address some of health care’s greatest challenges, including aging populations living with long-term conditions, persistent variations in the quality of care, and rising health care costs [1,2]. Although there is general agreement that HIT has the potential to improve safety, quality, and efficiency [3], large-scale HIT implementations require significant upfront investment, benefits are likely to materialize slowly, and those who put in most of the effort are often not those who benefit directly [4].

Such social and organizational challenges, which vary across contexts and technological functionalities, are often hard to navigate and predict for those managing change [5,6]. Despite efforts to identify success factors to guide implementation efforts, there is no simple recipe for success [7].

Formative evaluations can help to navigate these challenges. They can assist decision makers in moving from reactive to proactive strategies and identifying appropriate metrics to establish baselines and measure progress. In addition, they can help decision makers learn lessons more rapidly within the time frame of a project life cycle [8-10]. Formative evaluations can identify emerging unintended consequences and thereby, for instance, help to avert potential adverse outcomes for patient safety (eg, those arising from shortcomings in design, implementation strategy, and work practices) [11,12]. Evaluators should ideally work closely with strategic decision makers to keep projects on track and identify potential risks and mitigation strategies as early as possible.

However, despite these potential benefits, there is limited expertise in health services in conducting such formative evaluations. Existing organizational implementation evaluations, if conducted at all, often take place after an implementation has gone wrong, and use suboptimal methodologies.

Evidence-based frameworks to guide organizations in conducting HIT implementation evaluations have the potential to be helpful. A number of health informatics scholars have recently recognized this gap and developed various frameworks, some of which are summarized in Table 1 [13-19]. Some factors, such as user engagement and leadership, are well established in the change management literature. Other factors, such as political and market dimensions, have more recently received increasing recognition in shaping HIT implementations. The proliferation of frameworks poses a challenge for implementers seeking to navigate the literature and this paper seeks to integrate these frameworks.

**Table 1:** Examples of existing health information technology evaluation frameworks.

Framework	Key characteristics	Reference
Nonadoption, abandonment, scale-up, spread, and sustainability (NASSS) framework	This framework includes the following domains: the condition or illness, the technology, the value proposition, the adopter system, the organization(s), the wider context, and the interaction and mutual adaptation between all these domains over time.	Greenhalgh et al [13]
Framework for Evaluation of Informatics Tools	This framework includes the following stages: specification and needs requirements, component development, integration of system into a clinical setting, and routine use of a system.	Kaufman et al [14]
Health Information Technology Evaluation Toolkit	This framework includes the following dimensions: articulating goals of the project, understanding stakeholders, and benefits measurement.	Cusack and Poon [15]
Health Information Systems: human, organization, and technology-fit factors (HOT-fit)	This framework focuses on the fit between technological, human, and organizational dimensions.	Yusof et al [16]
Health Information Technology Reference-based Evaluation Framework (HITREF)	This framework includes 6 dimensions: structural quality, functional quality, effects on quality processes, effects on outcome quality of care, unintended consequences, and barriers and facilitators.	Sockolow et al [18]

Based on over 10 years of experience, we set out to update current thinking about formative evaluation frameworks. Drawing on the existing literature, our aim was to develop and apply a formative evaluation framework for HIT implementations that would offer guidance for implementers. In this study, we will present our experiential conclusions and highlight interrelationships between identified dimensions.

## Methods

### Description of the Data Set

We have led a series of qualitative, theoretically informed case studies of different HIT implementations in the context of national formative evaluations. These included electronic health records (EHRs), clinical decision support (CDS) systems, and a combination of CDS and computerized physician order entry (CPOE) systems [4,20,21]. Our ongoing involvement as principal investigators and researchers in these various formative evaluations provided a platform for understanding HIT implementation challenges.

Table 2 shows our data set, consisting of qualitative data collected between 2009 and 2018 by our research teams that included 11 social scientists. AS was the principal investigator on two of these projects [4,20], KC was the principal investigator on one [21], and RW was a senior adviser on all three [4,20,21]. We have published several papers from these evaluations, including both primary research and sets of evaluation recommendations based on the literature [4,20,21].

KC collected some primary data (approximately 100 interviews and 60 hours of observations on [20], 40 interviews on [4], and 14 interviews on [21]). The majority of case study sites (18 of 19) included hospital settings implementing EHR and CDS/CPOE functionality in the English National Health Service (NHS).

**Table 2:** Data set informing the development of the evaluation framework.

Project	Data set	Timeline
National evaluation of the implementation of electronic health records in secondary care in England	12 longitudinal qualitative case studies: 431 interviews, 590 hours of observations, 234 sets of field notes, and 809 documents	February 2009 to January 2011
National evaluation of the implementation of clinical decision support/computerized physician order entry systems in English hospitals	6 longitudinal qualitative case studies: 242 interviews, 32.5 hours of observations, and 55 documents	December 2011 to March 2016
National evaluation of a pilot decision support platform in Scottish primary care	30 interviews and 8 nonparticipant ethnographic observations	May 2018 to October 2018

### Sampling Overview

We defined a case as an organization implementing relevant functionality within the boundaries of an organizational setting. We sampled hospitals for maximum variation in relation to geographical location, size, implementation strategy, technological systems, and governance structures [22].

We sampled individual participants through a combination of convenience and snowball approaches with key local gatekeepers facilitating initial contacts [23]. Participants in case study settings included representatives with varying degrees of seniority from a range of clinical professions (medical, nursing, pharmacy, and allied health care professionals), managerial, and IT support staff. We also collected data from relevant policymakers and system vendors, in order to gain insights into the wider market and policy dynamics in which local implementations took place.

### Data Collection Overview

We collected data between February 2009 and October 2018. The majority of data consisted of digitally audio-recorded semistructured qualitative interviews (mainly face-to-face, some by telephone). These interviews explored expectations and experiences of implementing, using, and developing the new technology (depending on the background of the interviewee). Although interview guides varied with the specific focus functionality examined, key issues explored included the following: current systems, strategies, and organizational setup; views on potential system benefits and barriers to achieving these; and future directions and visions.

In many cases (18 of 19 case studies), we sought to interview participants longitudinally (ie, before the implementation of the system, during the implementation, and once they had time to get used to the HIT system).

Observations were nonparticipant in nature, opportunistic, and involved attending relevant strategic meetings (where the researcher took notes) or following a particular activity (eg, doctors using a specific system). Observations explored technological deployments in real-world contexts.

Documents consisted of minutes of strategic meetings, summaries of lessons learned, and business cases. These provided insights into planned activities and local narratives surrounding implementation.

### Development of the Framework

We began by conducting a review of existing frameworks and undertook a systematic literature review to explore which factors are important for the effective implementation of HIT [24]. The resulting prototype coding framework was iteratively refined over time and throughout projects. It included a number of dimensions and factors that formed the basis for coding qualitative data collected throughout case studies. In this process, we also allowed additional categories to emerge inductively [25]. Case studies were initially coded separately and then integrated iteratively across functionality (EHRs, CDS/CPOE in hospitals or what is known as ePrescribing in the United Kingdom, and CDS). This resulted in development of the prototype coding framework into a more comprehensive evaluation framework, which was synthesized to reflect the most pertinent categories and updated in light of the current literature. Here, our focus was on breadth rather than depth, aiming to produce a comprehensive overview of various stakeholder perspectives. We gave particular attention to stakeholders who were underrepresented (eg, vendors and administrative staff).

Our analysis and development of the novel framework was informed theoretically by the sociotechnical approach, structuration theory, the social shaping of technology, and the theory of the diffusion of innovations [26-29]. The final framework was agreed upon through iterative discussion.

## Results

**Figure 1:**
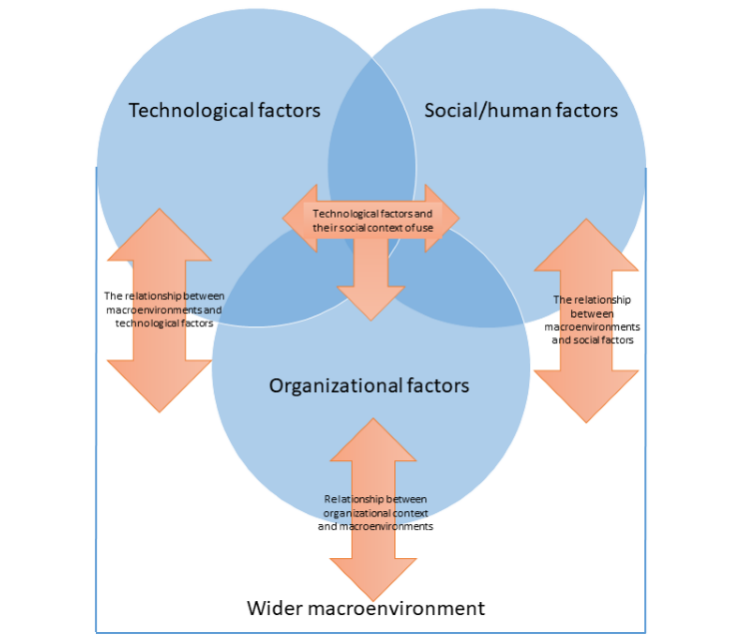
Diagram illustrating the evaluation framework.

We observed some commonalities across diverse settings and technological functions. The evaluation framework that has emerged from this work (Table 3, Figure 1) tackles important characteristics of the implementation landscape, where a range of technological, people (social/human), organizational, and wider macroenvironmental factors play an important role. Table 3 illustrates the key considerations in each of the dimensions. Figure 1 shows the interrelationship between the dimensions (technology, people, organizational, and macroenvironmental) and the various subcategories within each of these that need to be considered when implementing HIT.

**Table 3:** The Technology, People, Organizations, and Macroenvironmental factors (TPOM) framework, with example descriptions of dimensions.

Factor and dimension			Description
**Technological factors**			
	Usability	What is the ease of use and learnability of the technology?	
	Performance	Does the technology function as intended by developers?	
	Adaptability and flexibility	Can system design be changed to suit emerging needs?	
	Dependability	Is the system reliable and stable?	
	Data availability, integrity, and confidentiality	Is data in the system available, accessible, and usable for those who need it?	
	Data accuracy	Is the data in the system accurate?	
	Sustainability	Is use of the technology sustainable?	
	Security	Is the system secure?	
**Social/human factors**			
	User satisfaction	Who are the users? Are users satisfied with the technology?	
	Complete/correct use	Are features and functionality implemented and used as intended?	
	Attitudes and expectations	What benefits do users expect from using the technology and how can these be measured?	
	Engagement	Are users actively engaged in implementation, adoption, and optimization?	
	Experiences	Do users have negative experience with previous technologies?	
	Workload/benefits	Are the benefits and efforts relatively equal for all stakeholders?	
	Work processes	Does the system change relationships with patients, patterns of communication, and professional responsibilities (eg, increase of administrative tasks)?	
	User input in design	Is there effective communication between designers, information technology staff, and end users, as well as between management and end users?	
**Organizational context**			
	Leadership and management	Are management structures to support the implementation adequate?	
	Communication	Are aims, timelines, and strategy communicated?	
	Timelines	Are implementation timelines adequate?	
	Vision	What benefits do organizations expect from implementing the technology and how can these be measured? Is a coherent and realistic vision driving developments?	
	Training and support	Is the training adequate and realistic?	
	Champions	Are champions and boundary spanners utilized?	
	Resources	Is implementation adequately resourced? (includes technology, change management, and maintenance)	
	Monitoring and optimization	Is system performance and use monitored and optimized over time? Are lessons learned captured and incorporated in future efforts?	
**Wider macroenvironment**			
	Media	How is the technology viewed by the media and by the public? How does the organization view/manage media relations?	
	Professional groups	How is the technology viewed by professional groups?	
	Political context	What benefits do policymakers expect from the technology and how can these be measured? What is the national approach to achieving interoperability and does the system align with this? Is there a coherent vision, consistent approach, and a clear direction of travel, allowing a degree of local input?	
	Economic considerations and incentives	Are there clear incentives for organizations and users to implement? (eg, improvements in quality of care) Is sufficient funding in place to support the initiative?	
	Legal and regulatory aspects	Have legal and regulatory frameworks been established?	
	Vendors	Is vendor management effectively organized?	
	Measuring impact	Are various stakeholders working together to define, validate, test, and refine outcome measures and measurement strategies? Are outcome measures important, clinically acceptable, transparent, feasible, and usable?	

None of these dimensions outranks the others. The relationship between dimensions influences how implementation, adoption, optimization, and maintenance processes unfold over time. Below, we illustrate some examples of the interrelationship between the different overarching TPOM dimensions (technology, people, organizational, and macroenvironment; Figure 1). The dimensions identified are not intended to provide detailed support for the different components of the framework, but rather to illustrate the relationships and interdependencies of the framework components.

### Technological Factors and their Social Context of Use

We observed that, irrespective of the technology, systems needed to be usable, stable, and reliable (dependability), hold data securely (security), and only allow those with appropriate access rights to view confidential data (confidentiality). If systems and data within them were not dependable, users and implementers tended to lose confidence in their system choice.

…we are now questioning whether [name] is the right solution for our high secure service…

Manager, EHR Evaluation

However, it was also apparent that the design of technologies never occurred in isolation of the social and organizational context of use. This was exemplified through the many different ways in which technologies transformed how users worked in often unanticipated ways (eg, by making data entry more cumbersome; see the Social/human dimension, which includes “User satisfaction,” “Workload/benefits,” and “Work processes,” in Table 3), but also by how different technologies were optimized to suit organizational and user requirements over time (“Adaptability” in Table 3).

We had customized the system over a significant period of time to make it usable...

Clinical Lead, EHR Evaluation

System usability was the most important prerequisite for successful adoption. If users had to navigate a large number of interfaces and had difficulty finding relevant data, and if there was a general lack of intuitiveness, this slowed down their work (“Workload” and “Work processes” in Table 3).

All our doctors and nurses are having to work harder now, because we are having to see the same number of patients with less time, because you are spending more time on a computer now.

Consultant, EHR Evaluation

As a result, to not disrupt everyday delivery of care, staff had to employ workarounds (“Complete/correct use” in Table 3).

The staff just create workarounds of the system and some of them are ingenious and have gone on to change the system in a good way, but some aren’t as helpful.

Pharmacist, CDS/CPOE Evaluation

Although changes to system design could address these issues, modifications were in many instances cumbersome and lengthy (“Adaptability and flexibility” in Table 3). Vendors, in turn, struggled to find a balance between tailoring of applications to local needs (achieved through local configuration) and developing commercially viable generic system versions (“Vendors” in Table 3).

So when [vendor] deliver the product it will have a form designer so you will be able to go in the back end […]so the focus is to, for most of it make sure that it’s done through configuration not through software changes.

Manager, CDS/CPOE Evaluation

### The Relationship Between Organizational Context and Macroenvironments

Organizations tended to employ two distinct strategies to approach system implementation. These involved both “top-down” and “bottom-up” management approaches, as well as technology strategies that involved transformative change combined with automating existing processes. “Top-down” and “bottom-up” strategies occurred at two different levels. These included the following: (1) technology design/procurement (in the English National Programme for IT (NPfIT) characterized by centralized procurement; “Political context” in the Wider macroenvironment dimension); and (2) technology implementation strategy within the organization (“Leadership and management” in the Organizational dimension).

Balancing these tensions was instrumental for the perceived success of initiatives; this is not recognized in many existing frameworks. For example, although there was a perceived need for clinicians to be heavily involved in strategy, implementation, and deployment (“Communication” and “Vision”), stakeholders also recognized that some decisions at an organizational level had to satisfy the needs of diverse stakeholders, including those outside the immediate hospital environment (“Adaptability and flexibility” in Table 3).

Everybody wants their own changes so some of the changes are the [management] have decided to do it that way but that doesn’t mean Consultant A thinks that’s the right way, so there is often not acceptance.

Pharmacist, CDS/CPOE Evaluation

### The Relationship Between Macroenvironments and Technological Factors

It was very clear throughout our work that wider structures had a significant impact on organizational processes, ways of working, and technologies (see the Macroenvironmental dimension in Table 3). These included tensions between the long time frames needed to achieve transformation (5 to 10 years) and the episodic funding schemes (2- to 3-year programs; “Timelines” in Table 3). We also observed changing policy landscapes that involved a high turnover in senior staff and accompanying changes in visions of digital care and available funding (see “Political context” in Table 3). For example, when we began our work, England’s national strategy of implementing centrally procured systems had just started [30]. However, during the CDS/CPOE evaluation, there was an increasing focus on local involvement in decision making, driven by the demise of the NPfIT and increased economic pressures resulting from the global recession. When we completed data collection, there was again a growing recognition that national guidance was crucial for promoting implementation progress and interoperability [31].

I’ve got a concern that if one of those two parties come into power and it seems highly likely that they will, that the National Programme might be closed and [system] might be shut down and what then happens…do they close the whole of the National Programme in which case, do we go back to where we were eight years ago?

Manager, EHR Evaluation

In addition, we observed shifts in market and vendor structures and the technologies themselves. These are not sufficiently accounted for in existing frameworks (see “Vendors” in the Wider macroenvironment dimension in Table 3). During the NPfIT, we observed a limited number of large vendors pushing other players out of the English/UK market, although the dynamics changed after the demise of NPfIT and the termination of associated contracts. This gave way to a more vibrant vendor landscape (although the market is still not very open to new vendors), which has important implications for organizations and users as they can only procure technologies from those that are currently available.

With the breakdown of [NPfIT] we are now seeing a lot more [hospitals] looking to take advantage of electronic prescribing and we’re seeing an increased level of interest at this time to see if they can do this because effectively they’ve put the infrastructure in…

Vendor, CDS/CPOE Evaluation

We also saw how technologies were refined “in use” and through close working relationships between vendors and users over long periods (“Adaptability” in the Technology dimension in Table 3), which helped vendors/system designers overcome their limited knowledge of the social context of use. We have repeatedly seen the formation of vendor/user groups and observed how these helped to actively shape designs and markets (“User input in design”).

## Discussion

### Summary of Findings

We have developed an evaluation framework for implementers of HIT initiatives to guide implementation and optimization of functionality (Table 3). Although this draws on formative work, it can also guide summative evaluations. The TPOM framework includes key issues to consider in relation to technological, social/human, organizational, and wider macroenvironmental dimensions. Our ongoing work has shown that these dimensions are intimately related. Technologies never exist in isolation; it is therefore critical to appreciate that technological change will be accompanied by transformations in social groups, organizations, and the wider landscapes in which these are situated, including health policy, economic climates, and the development of markets.

### Strengths and Limitations

This work has drawn on a substantive composite qualitative data set collected over a long time frame. Therefore, it helped us to assess which dimensions were relatively stable over time, and only these were included in the TPOM framework. Insights presented here are views derived following careful critical reflections on a series of evaluations.

Although we acknowledge that many dimensions could be included, we deliberately attempted to keep the themes and subthemes manageable, thereby addressing a key issue in health technology evaluation: the usability of evaluation tools. We do not claim to capture all factors that play a role in implementation, adoption, and optimization of HIT, nor do we claim that our TPOM framework will provide a recipe for success. However, its pragmatic use in implementation and evaluation activity is likely to improve processes by prompting implementers to consider the most important dimensions influencing outcomes, thereby reducing unintended consequences and maximizing value. The framework now needs to be applied prospectively to confirm its utility across settings and regions. As such, we hope that it will provide a solid foundation for other countries to develop their own evaluation frameworks.

A key challenge faced by most existing evaluation frameworks, including ours, is that they neglect to account for the dynamic relationship between social and technological dimensions of change. As this relationship is a process, it does not lend itself well to presentation in 2D pragmatic evaluation tools. We have illustrated this dynamism in the Results section, drawing on concrete examples.

### Integration of Findings with the Current Literature

Many empirical studies of HIT implementation are primarily concerned with evaluating impact and therefore emphasize quantitative measurements guided by benefits realization frameworks [32,33]. Recent evaluation frameworks have expanded this limited focus to include a more in-depth appreciation of the interplay of social and technological factors shaping implementations. However, although acknowledging the complexity of the process, these nuanced frameworks tend to concentrate on one particular local and situated aspect of technology implementation, thereby neglecting the role of wider structuring conditions in shaping developments [16,34-37].

Others have considered wider structuring conditions, but the tools developed lack intuitiveness, usability, and practical applicability. For example, some frameworks that are designed to shed light on sociotechnical processes can become abstract and difficult to apply by those without academic backgrounds (which arguably includes the vast majority of those implementing change in health system settings) [38]. Others have become so complex that they may include a myriad of relevant dimensions, but this attempt to capture everything may result in a level of complexity that undermines the usefulness of the framework as a tool [13,26]. The challenge is to avoid Lewis Carroll’s cartographer’s dilemma of needing to make a comprehensive map on the same scale as the mapped landscape, which then no longer helps users navigate [39]. Our framework has sought to address this dilemma.

There are several commonalities with existing frameworks, including a recognition of key technology, human and organizational dimensions, and their interrelationship [13-16,18]. However, TPOM is not condition- or illness-specific [13]. It is not concerned with the likelihood of the technology being adopted and its spread [13], but it is designed to help implementers of technology consider how implementation is progressing, the potential emerging risks, and what aspects therefore need attention to facilitate adoption. Implementers can apply TPOM to any HIT project at any stage of implementation. It builds on other evaluation frameworks that take into account the microcontext of use [14-16,18], to include consideration of wider macroenvironment dimensions that influence implementation and adoption. It is not concerned with management tools, but with alignment of perspectives [15]. As such, it does not provide a “recipe for success”; rather, it is a tool designed to help implementers navigate a complex landscape with many conflicting agendas and considerations. When problems or risks are identified with the help of the framework, these can be systematically targeted to facilitate implementation and adoption.

### Policy Recommendations and Implications for Practice Emerging From This Work

Pragmatic formative evaluation frameworks can help to understand areas for potential improvement, benefits, and ways to streamline processes associated with technology implementation in health care settings. Evaluations need to move away from simple benefit realization approaches (that attempt to identify and measure benefits at the end of an implementation) toward formative evaluations that help the stakeholders involved adjust strategy along the way. Formative evaluations are part of a shift toward an evolutionary model where evaluation is a resource for faster and more effective learning.

Our proposed framework is a guide for implementers of technological change initiatives, to assist in planning, or during implementations of HIT initiatives in health care settings. We invite those who use it to suggest changes in both content and usability, as this will help to maximize its use and application. In due course, we hope to be able to draw on a range of data collected through the lens of the framework in different settings, refine it, and develop new insights in relation to each of the dimensions.

Large transformative policy programs aimed at facilitating technology implementation beyond hospitals are likely to require different evaluation frameworks, as their effects may be harder to trace and attribute.

### Conclusions

We have drawn on a substantial body of data to develop the TPOM framework (Table 3), which stakeholders can use to monitor change processes and, if necessary, adjust the direction of HIT implementation projects. Going forward, a key challenge is likely to be the ongoing tension between attempts to capture the dynamics, processes, and interrelationships involved in technological change; the large number of these dimensions and their complexity; and the usability of evaluation tools by those delivering care, which is linked to their potential to have impact. We encourage prospective application of the TPOM framework to determine its value.

## Abbreviations

CDS: clinical decision support
CPOE: computerized physician order entry
EHR: electronic health records
HIT: health information technology
NHS: National Health Service
NPfIT: English National Programme for IT
TPOM: Technology, People, Organizations, and Macroenvironmental factors

## References

[1] Blumenthal D (2010). Launching HITECH. N Engl J Med.

[2] Jha AK, Doolan D, Grandt D, Scott T, Bates DW (2008). The use of health information technology in seven nations. International Journal of Medical Informatics.

[3] Black AD, Car J, Pagliari C, Anandan C, Cresswell K, Bokun T, McKinstry B, Procter R, Majeed A, Sheikh A (2011). The Impact of eHealth on the Quality and Safety of Health Care: A Systematic Overview. PLoS Medicine.

[4] Cresswell KM, Bates DW, Williams R, Morrison Z, Slee A, Coleman J, Robertson A, Sheikh A, Avery T, Blake L, Chuter A, Slight SP, Girling A, Lee L, Lilford R, McCloughan L, Mozaffar H, Schofield J (2014). Evaluation of medium-term consequences of implementing commercial computerized physician order entry and clinical decision support prescribing systems in two 'early adopter' hospitals. Journal of the American Medical Informatics Association.

[5] May C, Mort M, Williams T, Mair F, Gask L (2003). Health technology assessment in its local contexts: studies of telehealthcare. Social Science & Medicine.

[6] Cresswell K, Sheikh A (2013). Organizational issues in the implementation and adoption of health information technology innovations: An interpretative review. International Journal of Medical Informatics.

[7] Cresswell KM, Bates DW, Sheikh A (2013). Ten key considerations for the successful implementation and adoption of large-scale health information technology. Journal of the American Medical Informatics Association.

[8] Catwell L, Sheikh A (2009). Evaluating eHealth Interventions: The Need for Continuous Systemic Evaluation. PLoS Medicine.

[9] Cresswell KM, Sheikh A (2014). Undertaking sociotechnical evaluations of health information technologies. Journal of Innovation in Health Informatics.

[10] Ammenwerth E, Rigby M (2016). Evaluation of implementation of health IT. Evidence-Based Health Informatics May 20.

[11] Cresswell KM, Mozaffar H, Lee L, Williams R, Sheikh A (2016). Workarounds to hospital electronic prescribing systems: a qualitative study in English hospitals. BMJ Quality & Safety.

[12] Mozaffar H, Cresswell KM, Williams R, Bates DW, Sheikh A (2017). Exploring the roots of unintended safety threats associated with the introduction of hospital ePrescribing systems and candidate avoidance and/or mitigation strategies: a qualitative study. BMJ Quality & Safety.

[13] Greenhalgh T, Wherton J, Papoutsi C, Lynch J, Hughes G, A'Court C, Hinder S, Fahy N, Procter R, Shaw S (2017). Beyond Adoption: A New Framework for Theorizing and Evaluating Nonadoption, Abandonment, and Challenges to the Scale-Up, Spread, and Sustainability of Health and Care Technologies. Journal of Medical Internet Research.

[14] Kaufman D, Roberts WD, Merrill J, Lai T, Bakken S (2006). Applying an Evaluation Framework for Health Information System Design, Development, and Implementation. Nursing Research.

[15] Cusack C, Poon E (2007). Rockville, MD: Agency for Healthcare Research and Quality Oct.

[16] Yusof MM, Kuljis J, Papazafeiropoulou A, Stergioulas LK (2008). An evaluation framework for Health Information Systems: human, organization and technology-fit factors (HOT-fit). International Journal of Medical Informatics.

[17] Campbell M, Fitzpatrick R, Haines A, Kinmonth A, Sandercock P, Spiegelhalter D, Tyrer P (2000). Framework for design and evaluation of complex interventions to improve health. BMJ.

[18] Sockolow PS, Bowles KH, Rogers ML (2015). Health Information Technology Evaluation Framework (HITREF) Comprehensiveness as Assessed in Electronic Point-of-Care Documentation Systems Evaluations. Stud Health Technol Inform.

[19] Andargoli AE, Scheepers H, Rajendran D, Sohal A (2017). Health information systems evaluation frameworks: A systematic review. International Journal of Medical Informatics.

[20] Sheikh A, Cornford T, Barber N, Avery A, Takian A, Lichtner V, Petrakaki D, Crowe S, Marsden K, Robertson A, Morrison Z, Klecun E, Prescott R, Quinn C, Jani Y, Ficociello M, Voutsina K, Paton J, Fernando B, Jacklin A, Cresswell K (2011). Implementation and adoption of nationwide electronic health records in secondary care in England: final qualitative results from prospective national evaluation in. BMJ.

[21] Cresswell K, Callaghan M, Mozaffar H, Sheikh A (2019). NHS Scotland's Decision Support Platform: a formative qualitative evaluation. BMJ Health Care Inform.

[22] Gentles S, Charles C, Ploeg J, McKibbon K (2015). Sampling in qualitative research: Insights from an overview of the methods literature. The Qualitative Report.

[23] Marshall MN (1996). Sampling for qualitative research. Family Practice.

[24] Cresswell K, Sheikh A (2009). The NHS Care Record Service (NHS CRS): recommendations from the literature on successful implementation and adoption. Journal of Innovation in Health Informatics.

[25] Fereday J, Muir-Cochrane E (2016). Demonstrating Rigor Using Thematic Analysis: A Hybrid Approach of Inductive and Deductive Coding and Theme Development. International Journal of Qualitative Methods.

[26] Sittig D, Singh H (2015). A new socio-technical model for studying health information technology in complex adaptive healthcare systems. Cognitive Informatics for Biomedicine.

[27] Stones R (2005). Structuration theory.

[28] Williams R, Edge D (1996). The social shaping of technology. Research Policy.

[29] Rogers EM (1995). Diffusion of innovations.

[30] Currie WL, Guah MW (2006). IT-Enabled Healthcare Delivery: The U.K. National Health Service. Information Systems Management.

[31] (2016). Department of Health and Social Care.

[32] Shang S, Seddon P (2000). A comprehensive framework for classifying the benefits of ERP systems. AMCIS Proceedings.

[33] Casey R, Wainwright D, Waring T (2015). Benefits realisation of information technology in the National Health Service: a paradigmatic review.

[34] Rippen HE, Pan EC, Russell C, Byrne CM, Swift EK (2013). Organizational framework for health information technology. International Journal of Medical Informatics.

[35] Nguyen L, Bellucci E, Nguyen LT (2014). Electronic health records implementation: An evaluation of information system impact and contingency factors. International Journal of Medical Informatics.

[36] Yen P, Bakken S (2011). Review of health information technology usability study methodologies. Journal of the American Medical Informatics Association.

[37] Lau F, Hagens S, Muttitt S (2007). A proposed benefits evaluation framework for health information systems in Canada. Healthcare Quarterly.

[38] Cornford T, Doukidis G, Forster D (1994). Experience with a structure, process and outcome framework for evaluating an information system. Omega.

[39] Carroll Lewis (1982). The Complete Illustrated Works.

